# Report on the Medical Department of the University of Penn. College

**Published:** 1851-10

**Authors:** 


*Report on the Medical Department of the University of Pennsylvania, for the Session* 1850–51, *to the Alumni of the School.* By the Medical Faculty.

The above pamphlet gives a statement of the flattering success attending the last session of this institution.

The matriculants during the last year numbered 468, and graduates 167, which might have been increased greatly if the faculty had relaxed the long established stringent rules of graduation. It is full, and much to the point, evincing a dignity becoming the standing of so old an institution. All interested in its progress and welfare will be highly interested in reading this short and ably written report.

